# Assessment of the effects of sugammadex on coagulation profiles using thromboelastographic parameters

**DOI:** 10.1038/s41598-020-68164-2

**Published:** 2020-07-07

**Authors:** Woon-Seok Kang, Hoyoung Lim, Byung-Soo Kim, Yeaji Lee, Kyung-Don Hahm, Seong-Hyop Kim

**Affiliations:** 1 Department of Anaesthesiology and Pain Medicine, Konkuk University Medical Center, Konkuk University School of Medicine, 120-1, Neungdong-ro (Hwayang-dong), Gwangjin-gu, Seoul, 05030 Republic of Korea; 2 Department of Anesthesiology and Pain Medicine, Asan Medical Center, University of Ulsan College of Medicine, Seoul, Republic of Korea; 3 Department of Infection and Immunology, Konkuk University School of Medicine, Seoul, Republic of Korea; 4 Research Institute of Medical Science, Konkuk University School of Medicine, Seoul, Republic of Korea

**Keywords:** Randomized controlled trials, Outcomes research

## Abstract

This study evaluated the effects of sugammadex at conventional doses of 2 and 4 mg/kg on the coagulation profile by analyzing thromboelastographic parameters and performing a traditional laboratory coagulation analysis. A total of 100 patients undergoing arthroscopic shoulder surgery were enrolled. The patients were randomly divided into the 2 mg and 4 mg groups. The laboratory coagulation test and thromboelastographic analysis were performed before and 15 min after administering sugammadex. Prothrombin time (PT) was significantly prolonged after sugammadex administration than before it in intragroup comparisons of the 2 mg group (12.8 ± 0.6 s vs. 13.6 ± 0.7 s, *p* < 0.001) and the 4 mg group (13.0 ± 0.5 s vs. 13.7 ± 0.5 s, *p* < 0.001). R time, derived from thromboelastography, was also significantly prolonged after sugammadex administration (4.7 ± 1.8 min vs. 5.8 ± 2.1 min, *p* = 0.005). In conclusion, the conventional doses of 2 or 4 mg/kg sugammadex prolonged PT. Sugammadex 4 mg/kg also prolonged R time, although the value was within the normal range. Therefore, physicians should be cautious with the higher sugammadex dose, particularly in patients with a high risk of bleeding because the higher dose was associated with less coagulation.

**Trial registration**: KCT0002133 (https://cris.nih.go.kr).

## Introduction

Sugammadex is a direct reversal agent for aminosteroid neuromuscular blocking agents. It does not affect cholinergic receptors and is expected to have fewer adverse side effects than traditional reversal agents, including the combination of acetylcholinesterase inhibitors and antimuscarinic agents^1,2^. There are few reports regarding the adverse effects of sugammadex, except for hypersensitivity reactions^3,4^. However, concerns remain regarding coagulation-related adverse effects following the use of sugammadex. Previous studies indicated that bolus injections of sugammadex at doses of 4 and 16 mg/kg for the reversal of a neuromuscular blockade increased the prothrombin time (PT) and activated partial thromboplastin time (aPTT) by about 10–29% compared to baseline within 30 min^5,6^. A 3–5.5% prolongation of PT and aPTT 10 min after 4 mg/kg sugammadex was administered was also reported^7^. However, doses of 4 and 16 mg/kg are higher than that conventionally used for the reversal of a neuromuscular blockade. Sugammadex is administered at a dose of 4 mg/kg for the reversal of a deep neuromuscular blockade, and a dose of 16 mg/kg is used only in emergency situations^8^. However, a moderate neuromuscular blockade is sufficient to maintain the surgical conditions for the typical case, and the dose of sugammadex to reverse moderate neuromuscular blockade is 2 mg/kg. Although neuromuscular transmission (NMT) monitoring is essential for general anesthesia with neuromuscular blockade, neuromuscular blocking agents have still been administered too frequently without NMT monitoring^9,10^. This results in the need for a higher dose of sugammadex to reverse the neuromuscular blockade. Moreover, a higher dose of sugammadex is occasionally administered with the expectation of a faster and more complete reversal of neuromuscular blockade. Thus, it was assumed that 2 and 4 mg/kg sugammadex were the doses typically used for moderate neuromuscular blockade.

We hypothesized that the conventional dose of sugammadex may affect a patient’s coagulation profile. This study evaluated the effects of sugammadex at the doses of 2 and 4 mg/kg for reversal of moderate neuromuscular blockade on patient coagulation profiles based on an analysis of thromboelastographic parameters and traditional laboratory coagulation analysis.

## Methods

### Study population

All experiments were performed following relevant guidelines and regulations. After obtaining approval from the Institutional Review Board of Konkuk University Medical Center, Seoul, Korea (KUH 1160095; November, 2015), registration at https://cris.nih.go.kr (KCT0002133, principal investigator: Seong-Hyop Kim, date of registration: November 4, 2016), and informed consent at an anesthesia pre-visit, patients undergoing arthroscopic shoulder surgery were evaluated prospectively at the university teaching hospital from November 2016 to April 2017. The exclusion criteria were as follows: (1) urgent or emergency cases, (2) patient age < 19 years or > 80 years, (3) reduced left and right ventricular function (ejection fraction < 40%), (4) previous respiratory disease, (5) severe hepatic disease, (6) decreased renal function (serum creatinine level more than double the normal range, urine output < 0.5 mL/kg/h or glomerular filtration rate < 60 mL/h), (7) preoperative anticoagulation medication, (8) previous neuromuscular disease, (9) family history of malignant hyperthermia, and (10) allergy to neuromuscular blocking agents or sugammadex. The study was performed in a prospective, randomized, double-blinded, and parallel manner (allocation ratio = 1:1). The patients were randomly assigned to the sugammadex 2 mg group (2 mg/kg group) or sugammadex 4 mg/kg group (4 mg group). For participant allocation, permuted block randomization was carried out by an institutional statistician using a computer-generated list of random numbers and sealed envelopes. Surgeons and nurses involved in patient care were aware that the study was being conducted but were blinded to the patient allocation and anesthetic regimen, including the laboratory measurements. The surgical procedure was performed by one surgeon and a single surgical team using the same method.

### Anesthetic regimen

The anesthetic regimen followed the protocol used in a previous study^11^. Anesthesia was induced and maintained by the attending anesthesiologist using a standard regimen. After establishing routine invasive systemic arterial blood pressure and non-invasive patient monitoring [pulse oximetry, electrocardiography, and bispectral index (BIS) measurement], anesthesia was induced following the administration of lidocaine (0.5 mg/kg) to reduce pain induced by propofol. Propofol (1.5 mg/kg) was administered intravenously to induce anesthesia and remifentanil (0.2 µg/kg/min) was continuously administered and maintained until the end of surgery. Rocuronium (0.6 mg/kg) was administered for muscle relaxation after loss of consciousness under the guidance of peripheral neuromuscular transmission (NMT) monitoring. Tracheal intubation was performed at a train-of-four (TOF) count of 0. In both groups, rocuronium was continuously infused after a 15 min bolus injection for tracheal intubation. The rocuronium infusion rate was adjusted to obtain a TOF count of 1 or 2 in both groups. During anesthesia maintenance, remifentanil was fixed at 0.2 µg/kg/min, and sevoflurane was adjusted to maintain the BIS between 40 and 60. The following ventilator (ADU; Datex-Ohmeda, Helsinki, Finland) settings were used in both groups: 4 L/min, consisting of air (3 L/min) and oxygen (1 L/min); the tidal volume was adjusted to achieve a tidal volume calculated as the ideal body weight × 8 mL; the respiratory rate was controlled using the end-tidal carbon dioxide pressure (EtCO_2_) ranging from 35 to 40 mmHg through capnography (S/5 Compact Anesthesia Monitor; Datex-Ohmeda); and no positive end-expiratory pressure (PEEP) with an inspiratory/expiratory ratio of 1:2. After anesthesia induction, the patient was changed from the supine position to the beach chair position and the surgical procedure was started. Vasoactive agents were used to maintain mean arterial blood pressure > 60 mmHg with systolic blood pressure < 180 mmHg and heart rate of 40–110 beats/min. Crystalloid solution (Plasma solution A Inj.^®^; CJ HealthCare, Seoul, Korea) was administered according to fluid maintenance requirements, redistribution, and evaporative surgical fluid losses based on body weight (4 mL/kg/h). After the end of the surgery, the patient was changed to the supine position, and all anesthetics were stopped. The lung recruitment maneuver was applied before the patient emerged from anesthesia to improve oxygenation and prevent atelectasis. Residual neuromuscular paralysis was antagonized by sugammadex at 2 mg/kg in the 2 mg group or 4 mg/kg in the 4 mg group under the guidance of NMT monitoring. Tracheal extubation was performed after confirming sufficient recovery (TOF ratio > 90%; BIS > 80, ability to open the eyes, ability to obey the anesthesiologist’s verbal commands, and ability to maintain a regular breathing pattern). The patient was then transferred to the post-anesthesia care unit (PACU). After confirmation of anesthesia recovery, the patient was transferred to the general ward. Postoperative care in the general ward was managed by the orthopedic surgeon with the institutional protocol.

### Monitoring of NMT

NMT monitoring was established and continuously monitored using TOF-Watch SX^®^ (Organon, Dublin, Ireland) with TOF stimulation according to the Good Clinical Research Practice guidelines for neuromuscular blocking agents^12^. A hand adapter with a thumb clip for preload was attached to the hand using an elastic band and adhesive tape.

The attending anesthesiologist opened the sealed envelope after the induction of anesthesia and before changing the position of the patient. To maintain the correct position of the transducer and avoid interference, the hand and wrist used for NMT monitoring were fixed with a splint, except for the thumb.

All NMT monitoring data were saved on a personal computer using TOF-Watch SX^®^ Monitor Software Version 2.2 (Organon). The skin temperature of the hand was measured and maintained above 32 °C. The central temperature was continuously monitored at the lower esophagus and kept above 35.5 °C.

### Measurements

All data were measured and recorded by one trained nurse who did not participate in patient care. The measured parameters were as follows: (1) PT (s), aPTT (s), and platelet counts (10^3^/μL) derived from laboratory blood tests; and (2) R time (min), K time (min), alpha angle (°), maximum amplitude (MA, mm), lysis 30 min (LY30, %), and coagulation index (CI) determined by thromboelastography (TEG). The parameters were measured after the surgical procedure and before sugammadex administration by blood sampling from the invasive arterial blood pressure monitoring catheter and 15 min after sugammadex administration in the PACU. A 3-mL blood sample was obtained and collected in a tube containing sodium citrate. The specimen was acclimated at room temperature or 37 °C for 15 min before placing 1 mL into a kaolin-containing tube for TEG. A 340-μL aliquot of the blood/kaolin mixture was combined with 20 μL of calcium chloride and analyzed immediately. TEG was conducted using a TEG^®^ 5000 Thromboelastography^®^ Hemostasis Analyzer system (Haemonetics, Braintree, MA). All reagents were purchased from Haemonetics. R time is the time interval from start to initiation of fibrin formation in minutes. K time is the time to achieve a determined clot strength in minutes. Alpha angle is the rate of clot formation in degrees. MA is the ultimate strength of the fibrin clot in mm. LY-30 is the clot resolution or fibrinolysis in percent lysis. CI is the coagulation index that is synthesized by the thromboelastographic parameters^13^.

Anesthesia duration with extubation time were checked at the end of anesthesia. Extubation time was defined as the time from sugammadex administration to extubation.

### Statistics

The primary outcome variables were the values of R time, K time, alpha angle, and MA. From a pilot study of 10 patients, R time of 5.45 ± 2.32 min (normal value, 2–8 min), K time of 1.84 ± 0.58 min (normal value, 1–3 min), alpha angle of 66.4° ± 5.4° (normal value, 55°–78°), and MA of 64.7 ± 5.8 mm (normal value, 51–69 mm) were recorded before sugammadex administration. A minimum detected difference of 20% before and after administration of 2 or 4 mg/kg of sugammadex was considered clinically significant, because 20% prolongation of each parameter for TEG was regarded as hypocoagulation status and the patient had the risk of bleeding. Sample sizes of 50 for R time, 30 for K time, four for alpha angle, and five for MA in each group were calculated to be appropriate to achieve a power of 0.9 and an α value of 0.05.

Statistical analyses were performed using SigmaStat software (ver. 3.1; SYSTAT Software, San Jose, CA). Continuous variables were analyzed using Student’s *t* test for intergroup comparisons, paired *t* test after the normality test (Kolmogorov–Smirnov method) or Wilcoxon’s Signed Rank test for intragroup comparisons. Categorical variables were analyzed using the chi-squared test. Data are expressed as the numbers of patients, and means ± SD or medians (25–75%, interquartile range). In all analyses, *p* < 0.05 was taken to indicate statistical significance.

## Results

**Figure 1:**
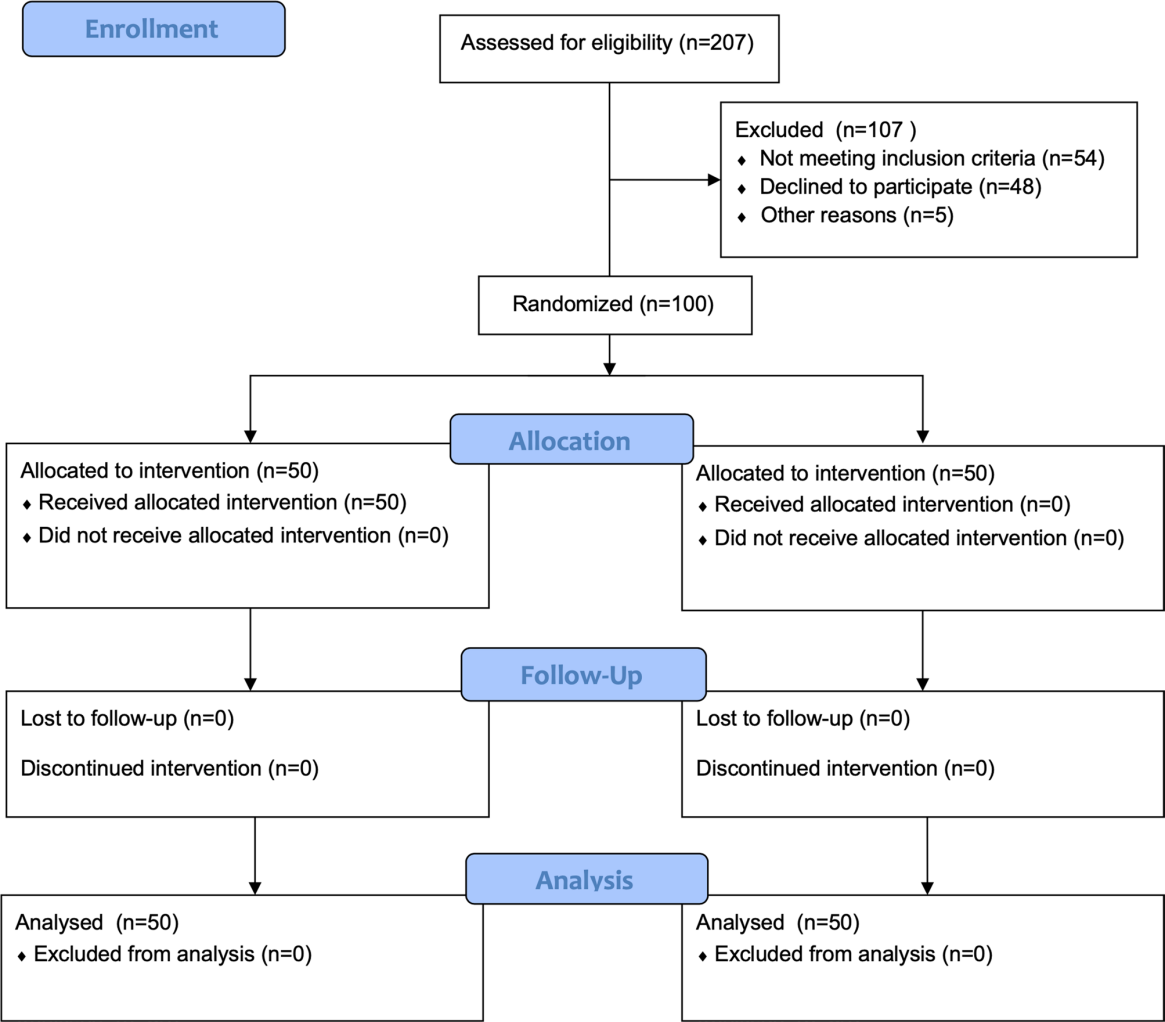
CONSORT flow diagram.

During the study, 207 arthroscopic shoulder surgeries were performed, and 100 patients were eligible for the study. Overall, 107 patients were excluded: 48 for refusal to participate in the study; 35 for previous respiratory disease; 19 for preoperative dysrhythmia; and 5 due to instrument error. Therefore, a total of 50 patients were included in each group (Fig. 1). The study was terminated once the planned sample size was attained. No harmful results or unintended events due to the investigation occurred in the study groups.

The patients’ demographic profiles were similar in both groups, including anesthesia duration with extubation time (Table 1).

**Table 1:** Demographic and perioperative parameters.

	2 mg group	4 mg group	*p* value
Age (years)	57 (50–64)	61 (56–63)	0.232
Gender (M/F)	32/18	33/17	0.834
Height (cm)	164 (159–168)	165 (157–170)	0.579
Weight (kg)	69 ± 12	68 ± 11	0.568
Anesthesia time (min)	139 (115–160)	136 (115–160)	0.733
Extubation time (min)	5 (3–7)	4 (3–6)	0.743

Data are expressed as numbers of patients, mean ± standard deviation or median (25–75% interquartile range).

In intragroup comparisons of the 2 mg group, the values of PT before and after sugammadex administration were significantly different and the value after sugammadex administration was significantly prolonged (12.8 ± 0.6 s vs*.* 13.6 ± 0.7 s, difference: 0.8 [95% confidence interval (CI) 0.6–1.0] s, *p* < 0.001). The other parameters derived by conventional coagulation and thromboelastographic profiles were not different (Table 2).

**Table 2:** Coagulation profiles and thromboelastographic parameters between sugammadex 2 mg versus 4 mg.

	2 mg group			4 mg group		
	Before	After	*p *value	Before	After	*p *value
PT (s)	12.8 ± 0.6	13.6 ± 0.7	< 0.001	13.0 ± 0.5	13.7 ± 0.5	< 0.001
aPTT (s)	34.6 ± 3.7	34.1 ± 3.5	0.410	35.5 (34.0 to 38.0)	35.4 (32.7 to 38.6)	0.848
PLT (10^3^/µL)	243 ± 62	242 ± 64	0.536	238 ± 49	239 ± 49	0.964
R time (min)	4.5 ± 2.3	5.2 ± 2.1	0.066	4.7 ± 1.8	5.8 ± 2.1	0.005
K time (min)	1.5 (1.3 to 1.8)	1.6 (1.1 to 2.2)	0.894	1.6 (1.2 to 1.9)	1.5 (1.2 to 1.8)	0.615
Alpha angle (°)	63.7 ± 9.3	63.5 ± 10.9	0.910	66.4 (62.0 to 73.2)	66.8 (64.7 to 71.3)	0.761
MA (mm)	67.5 (63.9 to 71.6)	65.6 (62.1 to 72.0)	0.585	64.8 (60.4 to 72.0)	65.4 (62.8 to 71.9)	0.995
LY30 (%)	0.2 (0.0 to 1.4)	0.3 (0.0 to 1.1)	0.790	0.3 (0.0 to 1.0)	0.2 (0.0 to 0.7)	0.627
CI	1.5 (− 0.1 to 3.0)	2.1 (− 0.7 to 3.5)	0.426	1.6 ± 2.4	0.8 ± 2.3	0.105

Data are expressed as mean ± standard deviation or median (25–75% interquartile range).

*PT* prothrombin time, *aPTT* activated partial thromboplastin time, *PLT* platelet, *MA* maximum amplitude, *LY* lysis, *CI* coagulation index.

In intragroup comparisons of the 4 mg group, the values of PT before and after sugammadex administration were significantly different and the value after sugammadex administration was significantly prolonged (13.0 ± 0.5 s vs*.* 13.7 ± 0.5 s, difference: 0.7 [95% CI 0.6–0.8] s, *p* < 0.001). The R time values derived by TEG were significantly different between before and after sugammadex administration and the value was significantly longer in comparison to before sugammadex administration (4.7 ± 1.8 min vs*.* 5.8 ± 2.1 min, difference: 1.1 [95% CI 0.4–2.0] min, *p* = 0.005). There were no differences in the other parameters (Table 2).

**Figure 2:**
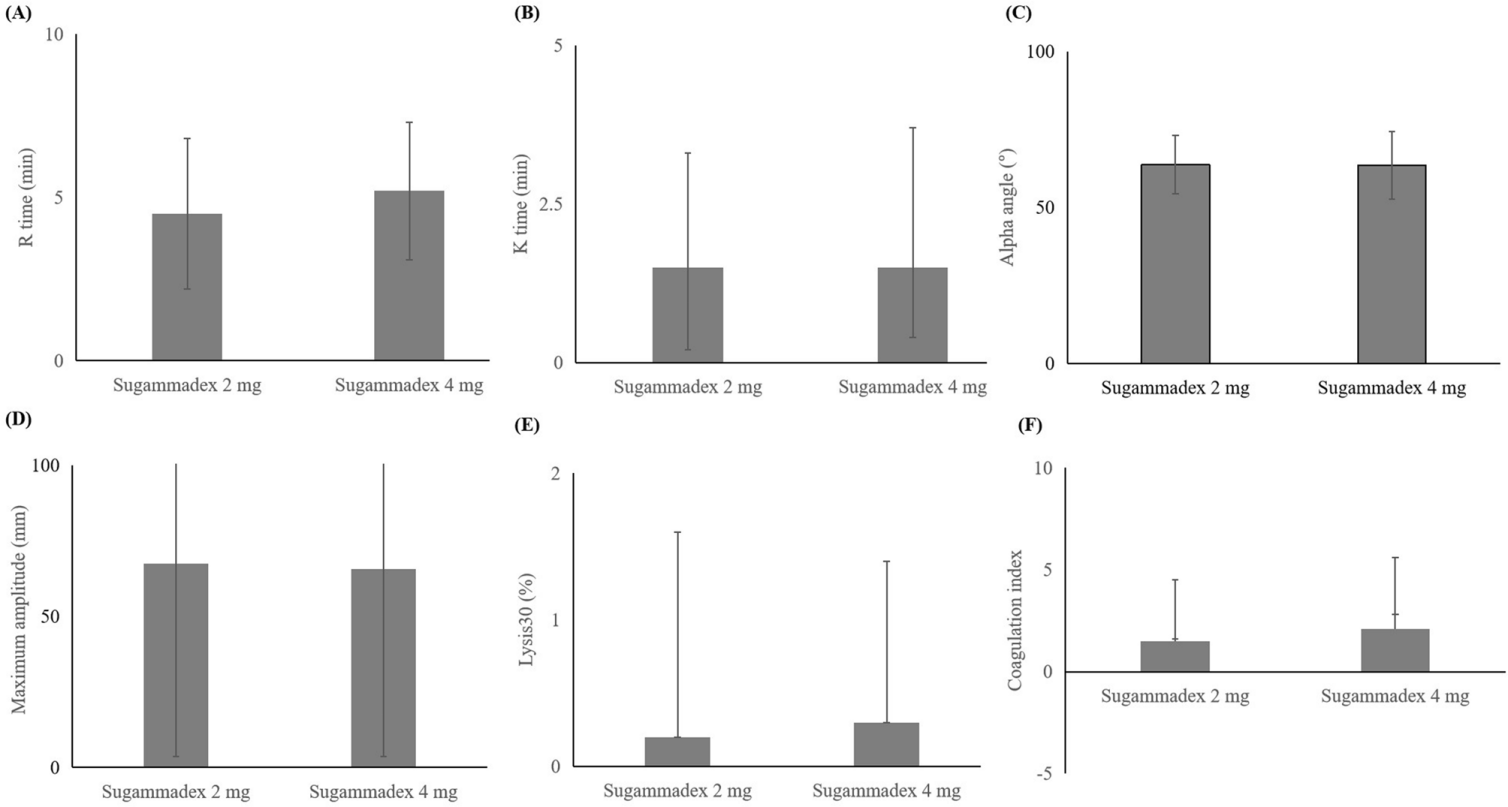
Thromboelastographic parameters after sugammadex 2 mg versus 4 mg. R time had the *p* value = 0.672. K time had the *p *value = 0.548. Alpha angle had the *p* value = 0.292. Maximum amplitude had the *p* value = 0.804. Lysis30 had the *p* value = 0.225. Coagulation index had the *p* value = 0.858.

In intergroup comparisons between groups according to measurement time (before and after sugammadex administration), there were no differences in any of the conventional coagulation and thromboelastographic profiles (Table 2 and Fig. 2).

## Discussion

Our results demonstrate that among traditional conventional laboratory coagulation tests, including PT, aPTT, and platelet count, only PT was prolonged after the administration of sugammadex at doses of 2 and 4 mg/kg. Among the TEG parameters, sugammadex at a dose of 4 mg/kg also increased R time, although no such effect was observed at a sugammadex dose of 2 mg/kg. However, the prolonged values of PT and R time were within the respective normal ranges.

Several studies have reported the prolongation of PT or aPTT in association with the use of sugammadex^5–7^. However, traditional conventional laboratory coagulation analysis has several limitations, including the lack of assessment of dynamic whole blood coagulation (e.g., coagulation strength). Therefore, additional studies are required to confirm the effects of sugammadex on coagulation using qualitative methods. We also obtained prolongation of PT, one of the traditional conventional laboratory coagulation parameters, after sugammadex administration at doses of 2 and 4 mg/kg. However, the degree of prolongation was just 0.8 s for 2 mg/kg and 0.7 s for 4 mg/kg, and the values of PT after the administration of sugammadex were within the normal range. The R time from TEG was prolonged after the administration of 4 mg/kg sugammadex in the present study. The prolongation of R time indicates the dysfunction of coagulation factors in the early phase of the coagulation cascade and can induce a hypocoagulative status^8^. The prolonged R time caused by 4 mg/kg sugammadex in the present study might indicate the initiation of coagulation dysfunction, although the R time of 4 mg/kg sugammadex was within the normal range without any significant intergroup difference. Our results correspond well to previous studies on the relationship between the coagulation profile and sugammadex^14,15^. Carron et al*.* researched the effects of sugammadex on coagulation profiles in morbidly obese patients using rotational thromboelastometry and their results showed a slight increase in clotting time^14^. In their study, a regression analysis also showed a positive relationship between sugammadex dose and clotting time. Lee et al. conducted an in vitro study on the effect of sugammadex on TEG. They used three different concentrations of sugammadex, corresponding to the maximal plasma concentrations after administering 4, 16, and 32 mg/kg sugammadex to healthy subjects. They reported that the higher sugammadex concentrations were significantly associated with reduced coagulation, as evidenced by the thromboelastographic parameters^15^. Although we checked only the 2 and 4 mg/kg doses of sugammadex, a greater prolongation of R time is possible following the administration of a higher dose of sugammadex (e.g., 8 or 16 mg/kg), according to results of previous studies^14,15^ and our study. Therefore, we speculate that the relationship between sugammadex and R time is does-dependent and that the higher sugammadex dose may be associated with reduced coagulation, although we did not run a regression analysis between sugammadex dose and the thromboelastographic parameters.

The present study had two limitations. First, it only evaluated the effect of sugammadex on coagulation profiles. The ultimate goal of a coagulation test is to predict or confirm whether a patient has a tendency to bleed. Therefore, a coagulation profile with postoperative bleeding volume is informative and meaningful. However, we did not confirm these parameters. Measuring the exact postoperative bleeding volume without any bias or error is difficult. Intra- or postoperative management, including surgical technique, drugs, and fluids, affect the coagulation profile and bleeding volume. If surgery with a high-risk of bleeding was chosen for the study, the effect of sugammadex on the coagulation profile with postoperative blood loss and related complications would be noticeable. However, the intervention or management of bleeding could be a confounding factor, hindering the interpretation of the pure effect of sugammadex on the coagulation profile. Therefore, we enrolled healthy patients, without any risk of bleeding, undergoing arthroscopic shoulder surgery, with a low-risk of bleeding to minimize interruptions in intraoperative management (standardization of intraoperative management, including drugs and fluids). Second, the degree of neuromuscular blockade was moderate, not deep, in the present study, and 4 mg/kg sugammadex was administered for moderate neuromuscular blockade. It is not clear whether the excessive dose of sugammadex resulted in the prolongation of PT and R time. Further study of the effects of higher-dose sugammadex administered appropriately for neuromuscular blockade on coagulation would provide more definitive information.

In conclusion, the conventional doses of 2 or 4 mg/kg sugammadex prolonged PT. Sugammadex 4 mg/kg also prolonged R time, derived by TEG, although the value was within the normal range. Therefore, the higher sugammadex dose should be used cautiously, especially in patients with a high risk of bleeding, because it was associated with reduced coagulation.

## References

[1] Jones RK, Caldwell JE, Brull SJ, Soto RG (2008). Reversal of profound rocuronium-induced blockade with sugammadex: a randomized comparison with neostigmine. Anesthesiology.

[2] Blobner M (2010). Reversal of rocuronium-induced neuromuscular blockade with sugammadex compared with neostigmine during sevoflurane anaesthesia: results of a randomised, controlled trial. Eur. J. Anaesthesiol..

[3] Miyazaki Y (2018). Incidence of anaphylaxis associated with sugammadex. Anesth. Analg..

[4] Min KC (2018). Hypersensitivity incidence after sugammadex administration in healthy subjects: a randomised controlled trial. Br. J. Anaesth..

[5] De Kam PJ (2014). Effects of sugammadex on activated partial thromboplastin time and prothrombin time in healthy subjects. Int. J. Clin. Pharmacol. Ther..

[6] Dirkmann D (2016). Anticoagulant effect of sugammadex: just an in vitro artifact. Anesthesiology.

[7] Rahe-Meyer N (2014). Effect of reversal of neuromuscular blockade with sugammadex versus usual care on bleeding risk in a randomized study of surgical patients. Anesthesiology.

[8] Fuchs-Buder T, Meistelman C, Raft J (2013). Sugammadex: clinical development and practical use. Korean J. Anesthesiol..

[9] Phillips S, Stewart PA, Bilgin AB (2013). A survey of the management of neuromuscular blockade monitoring in Australia and New Zealand. Anaesth. Intensive Care.

[10] Naguib M, Kopman AF, Lien CA, Hunter JM, Lopez A, Brull SJ (2010). A survey of current management of neuromuscular block in the United States and Europe. Anesth. Analg..

[11] Kang WS (2020). Deep neuromuscular blockade during spinal surgery reduces intra-operative blood loss: A randomised clinical trial. Eur. J. Anaesthesiol..

[12] the Stockholm revision (2007). Fuchs-Buder T, Claudius C, Skovgaard LT, Eriksson LI, Mirakhur RK, Viby-Mogensen J; 8th International Neuromuscular Meeting. Good clinical research practice in pharmacodynamic studies of neuromuscular blocking agents II. Acta Anaesthesiol. Scand..

[13] Whiting D, DiNardo JA (2014). TEG and ROTEM: technology and clinical applications. Am. J. Hematol..

[14] Carron M (2018). Effect of sugammadex on coagulation as detected by rotational thromboelastometry in morbidly obese patients. Minerva Anestesiol..

[15] Lee IO, Kim YS, Chang HW, Kim H, Lim BG, Lee M (2018). In vitro investigation of the effects of exogenous sugammadex on coagulation in orthopedic surgical patients. BMC Anesthesiol..

