# Author Correction: Assessment of the effects of sugammadex on coagulation profiles using thromboelastographic parameters

**DOI:** 10.1038/s41598-020-76583-4

**Published:** 2020-11-10

**Authors:** Woon-Seok Kang, Hoyoung Lim, Byung-Soo Kim, Yeaji Lee, Kyung-Don Hahm, Seong-Hyop Kim

**Affiliations:** 1 Department of Anaesthesiology and Pain Medicine, Konkuk University Medical Center, Konkuk University School of Medicine, 120-1, Neungdong-ro (Hwayang-dong), Gwangjin-gu, Seoul, 05030 Republic of Korea; 2 Department of Anesthesiology and Pain Medicine, Asan Medical Center, University of Ulsan College of Medicine, Seoul, Republic of Korea; 3 Department of Infection and Immunology, Konkuk University School of Medicine, Seoul, Republic of Korea; 4 Research Institute of Medical Science, Konkuk University School of Medicine, Seoul, Republic of Korea

Correction to: *Scientific Reports* https://doi.org/10.1038/s41598-020-68164-2, published online 07 July 2020

The original version of this Article contained an error in Affiliation 2, which was incorrectly given as ‘Department of Anesthesiology and Pain Medicine, Asan Medical Center, Ulasn University, Seoul, Republic of Korea’. The correct affiliation is listed below:

‘Department of Anesthesiology and Pain Medicine, Asan Medical Center, University of Ulsan College of Medicine, Seoul, Republic of Korea’

This error has now been corrected in the HTML and PDF versions of the Article.

